# Older Adults’ Outdoor Walking: Inequalities in Neighbourhood Safety, Pedestrian Infrastructure and Aesthetics

**DOI:** 10.3390/ijerph13121179

**Published:** 2016-11-25

**Authors:** Razieh Zandieh, Javier Martinez, Johannes Flacke, Phil Jones, Martin van Maarseveen

**Affiliations:** 1 Faculty of Geo-Information Science and Earth Observation (ITC), University of Twente, PO Box 217, 7500 AE Enschede, The Netherlands; j.martinez@utwente.nl (J.M.); j.flacke@utwente.nl (J.F.); m.f.a.m.vanmaarseveen@utwente.nl (M.v.M.); 2 School of Geography, Earth and Environmental Sciences, University of Birmingham, Edgbaston, Birmingham B15 2TT, UK; p.i.jones@bham.ac.uk

**Keywords:** physical activity, walking, deprivation, built environment, older adults, perception, inequalities, GPS

## Abstract

Older adults living in high-deprivation areas walk less than those living in low-deprivation areas. Previous research has shown that older adults’ outdoor walking levels are related to the neighbourhood built environment. This study examines inequalities in perceived built environment attributes (i.e., safety, pedestrian infrastructure and aesthetics) and their possible influences on disparities in older adults’ outdoor walking levels in low- and high-deprivation areas of Birmingham, United Kingdom. It applied a mixed-method approach, included 173 participants (65 years and over), used GPS technology to measure outdoor walking levels, used questionnaires (for all participants) and conducted walking interviews (with a sub-sample) to collect data on perceived neighbourhood built environment attributes. The results show inequalities in perceived neighbourhood safety, pedestrian infrastructure and aesthetics in high- versus low-deprivation areas and demonstrate that they may influence disparities in participants’ outdoor walking levels. Improvements of perceived neighbourhood safety, pedestrian infrastructure and aesthetic in high-deprivation areas are encouraged.

## 1. Introduction

Outdoor walking, as a type of physical activity, takes place in outdoor spaces. It has well-known benefits for health in later life [1,2] and older adults are recommended to take outdoor walks [3]. However, evidence shows that most older adults, especially those living in high-deprivation areas of cities (areas with high levels of social and economic disadvantages) [4,5], are physically inactive [6,7]. Fox, et al. [8] found that older residents of high-deprivation areas walk less than those of low-deprivation areas. This finding indicates the necessity of encouraging outdoor walking among older adults, especially those living within high-deprivation areas.

Although some previous studies have found insignificant relationships between the built environment and older adults’ walking [9,10], there is growing evidence showing that older adults’ walking level is related to the built environment of residential neighbourhoods [11,12,13]. Neighbourhood built environment may encourage older adults to take outdoor walks [1,3]. Built environment attributes fall into two categories [14]: (1) macro built environment attributes, including neighbourhood residential density, land use-mix and route connectivity, which shape the overall design and structure of a neighbourhood; and (2) micro built environment attributes, including safety, pedestrian infrastructure and aesthetics (defined in Table 1), which shape route characteristics in a neighbourhood [14,15]. Most previous studies on walking have focused on macro built environment attributes [16,17,18,19], however, examining micro built environment attributes may also be useful [14]. Micro built environment attributes can be modified at lower cost and more rapidly than macro built environment attributes [14,15,20]. Evidence indicates that neighbourhood safety, pedestrian infrastructure and aesthetics are important for supporting and encouraging outdoor walking [21,22,23,24,25] because pedestrians move slowly in outdoor spaces thus affording the ability to notice route characteristics [26]. These built environment attributes seem especially important for older adults’ outdoor walking [1,27,28,29]. Unfavourable neighbourhood safety, poor pedestrian infrastructure and unattractive aesthetics could blunt or negate the positive influences of macro built environment attributes on outdoor walking [15,30]. For example, older adults may avoid walking to available attractive destinations located in walking distances due to high risk of accident [28]. Evidence has shown that for older adults’ outdoor walking, maximising the neighbourhood aesthetics or quality of pedestrian infrastructure is more important than minimizing the distance to a destination [28].

Accordingly, findings on lower level of older adults’ outdoor walking in high- versus low-deprivation areas raises some questions related to the neighbourhood built environment: do older residents of high-deprivation areas have a less supportive neighbourhood built environment for outdoor walking compared to those living in low-deprivation areas? How do neighbourhood safety, pedestrian infrastructure and aesthetics influence older residents’ outdoor walking levels in low- and high-deprivation areas? These questions are crucial for urban planning, since this discipline aims to provide a built environment that encourages and supports all citizens’ outdoor walking [32,33]. From an urban planning perspective, these questions are put in the context of “spatial inequality”. Using the UN-Habitat report [34], spatial inequality (on a city scale) is defined as the uneven provision of urban opportunities and resources among different urban areas (e.g., slum and non-slum areas). Answering these questions helps urban planners to identify spatial inequalities in built environment support for older adults’ outdoor walking and to pinpoint shortcomings in high-deprivation areas.

To answer the questions outlined above, neighbourhood safety, pedestrian infrastructure and aesthetics can be measured either objectively or subjectively. Although built environment attributes can be measured objectively, they may not correspond to an individual’s own assessment of the built environment [35]. Bowling and Stafford [36] have found that objective and subjective measures of neighbourhood capture different environmental attributes which are independently related to physical and social functioning in older adults. Theories of behaviour change commonly employed in physical activity promotion suggest that subjectively measured (or perceived) built environment attributes may be more closely related to health behaviour than objectively measured built environment attributes [35,37]. The advantage of subjectively measured (or perceived) built environment attributes is that they reflect personal assessment of the built environment [2]. It is known that older adults are more sensitive to the built environment than younger adults [38]. Physical changes associated with the aging process may reduce older adults’ confidence or ability to handle their interactions with the built environment [39]. Therefore, for older adults, the same built environmental constraint (e.g., uneven pavement) may be assessed more challenging than for younger adults [40]. Thus, examining subjectively measured (or perceived) neighbourhood built environment attributes is important in studies on older adults’ walking.

Previous research has examined associations between perceived neighbourhood safety, pedestrian infrastructure and aesthetics, and outdoor walking level, but they have reported mixed results [27]. Some previous research on physical activity has also shown inequalities in perceived neighbourhood safety, pedestrian infrastructure or aesthetics in high- versus low-deprivation areas [41,42]. However, the influences of these spatial inequalities on older residents’ total outdoor walking levels have received less attention. Grant, et al. [43] have considered inequalities in walking conditions among older adults living in low- and high-deprivation areas without examining older adults’ total outdoor walking levels.

This study aims to examine inequalities in perceived neighbourhood safety, pedestrian infrastructure and aesthetics in high- versus low-deprivation areas and their possible influences on disparities in older residents’ total outdoor walking levels. For this purpose, it uses a mixed-method approach and addresses two research questions: (1) How unequal are perceived neighbourhood safety, pedestrian infrastructure and aesthetics in high- versus low-deprivation areas? and (2) What are the relationships between perceived neighbourhood safety, pedestrian infrastructure, aesthetics, and older residents’ outdoor walking levels?

These research questions are addressed quantitatively combined with a qualitative examination of the perceived influences of neighbourhood safety, pedestrian infrastructure and aesthetics on older residents’ outdoor walking levels in low- and high-deprivation areas. This study controls for older residents’ personal characteristics (e.g., age, gender, marital status and ethnicity) because evidence shows that people’s personal characteristics affect their walking levels [44,45,46].

## 2. Materials and Methods

This empirical study was conducted from July to November 2012 in Birmingham—a large, ethnically diverse city with more than one million residents in England [47]. The authors adopted a concurrent mixed-method design [48]. This research design helped to enrich the quantitative study (on “perceived neighbourhood built environment attributes and outdoor walking levels”) with qualitative information (on “how perceived neighbourhood built environment attributes may, in the view of older residents, influence outdoor walking levels”). Therefore, qualitative results were used to support and interpret the quantitative results. This study used Geographic Positioning System (GPS) technology to collect data on outdoor walking levels and used a paper questionnaire (containing closed and open-ended questions) and conducted walking interviews to collect data on perceived neighbourhood built environment attributes. Detailed information on data collection is presented in Section 2.3 and Section 2.4. This study received ethical approval from University of Birmingham’s Humanities and Social Sciences (HASS) Ethical Review Committee on 10 August 2012. The approval code is ERN_12-0874. All participants signed a consent form before participating in the study.

### 2.1. Selection of Low- and High-Deprivation Areas

**Figure 1:**
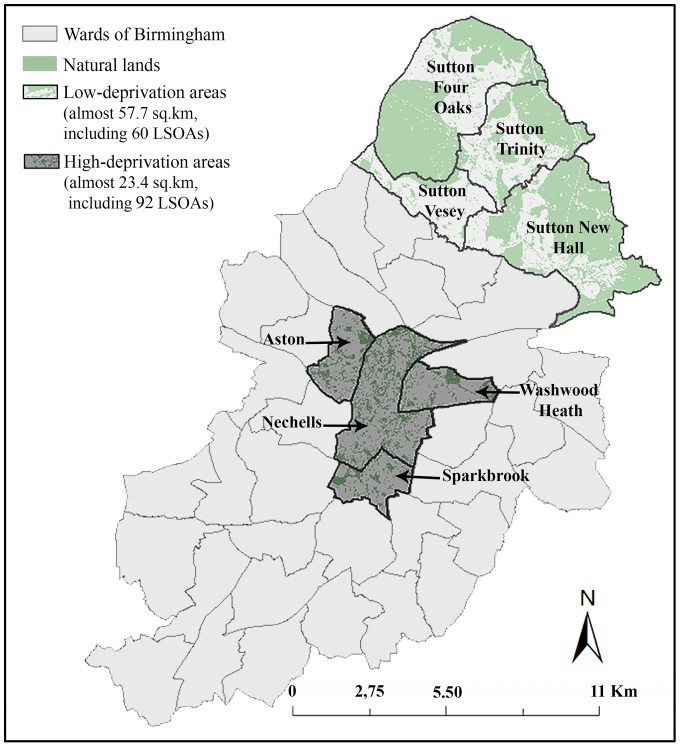
Locations of low- and high-deprivation areas in Birmingham. (OS open data Boundary-line ^©^ Crown copyright/database right 2012 and OS MasterMap data © Crown Copyright/database right 2012. An Ordnance Survey/EDINA Digimap supplied service.)

Low- and high-deprivation areas were identified on an electoral ward scale. For this purpose, the Index of Multiple Deprivation (IMD)—an aggregated score of deprivation across England [8,49] —was used. The IMD is published at Lower Layer Super Output Areas (LSOAs) level: the geographic areas that share similar populations of around 1500 persons [49,50]. LSOAs of Birmingham were classified by using the IMD to identify the 20% most deprived and 20% least deprived LSOAs in the city, which were then used to classify the city’s wards. A ward was identified as a relatively low- (or high-) deprivation area of city, if more than 50% of its area was covered by the least (or most) deprived LSOAs. In this way, four low-deprivation and four high-deprivation wards were identified in Birmingham (Figure 1). These wards were used for participant recruitment.

### 2.2. Participant Recruitment

A convenience sampling approach was used to recruit participants from all 8 selected wards. Participants were recruited from social centres (e.g., community centres, University of the Third Age, libraries, etc.) in low- and high-deprivation areas. Using a convenience sampling approach is often the norm in health behavior research on older adults [51]. Older adults were informed about the process of participation by posting advertisements and arranging information sessions in social centres. A translator explained the research to older adults who did not speak English.

Older adults (65 years and upward), resident of a low- or high-deprivation area, able to walk, independent in their daily life activities, and mentally healthy were eligible to participate in this research. Ability to speak English was not an eligibility criteria. A translator or an assistant helped participants (*n* = 58) who did not speak English or needed assistance in completing the questionnaire. Participants were screened for their ethnicity: to achieve minimum difference with ethnic composition in the total population of the selected wards, we used quota sampling and the proportions of different ethnicities identified in the last available census (2001) were mirrored in the sample.

In total, 216 participants received GPS tracking units. Participants (*n* = 43) who did not (or forgot to) use the GPS tracking unit were excluded and the final sample included 173 participants (93 and 80 participants from low-and high-deprivation areas, respectively).

A sub-sample (nine participants from low-deprivation areas and 10 participants from high- deprivation areas, limited by time and cost considerations) was drawn from the main sample. The participants were recruited from different parts of low- and high-deprivation areas and depending on participants’ willingness and availability for participating in walking interviews. All participants spoke English. To achieve maximum similarity with the total sample, the researchers considered ethnic differences between low- and high-deprivation areas and used quota sampling for recruiting of participants for the sub-sample.

### 2.3. Measuring Outdoor Walking Level with a GPS Tracker

Following suggestions from previous studies [52,53,54,55,56], GPS technology was used for measuring outdoor walking level objectively. In this way, data on date, time and location (x, y) of 173 participants’ outdoor walking were collected. This technology provides accurate data on time and location (e.g., parks and streets) of outdoor walking [56,57] without the recall bias issues related to diaries [58].

A GPS tracking unit (i-gotU GT-600 GPS data-logger, Mobile Action Technology Inc., New Taipei City, Taiwan), which had been used in previous research [59,60], was used in this study. This device is adequate to use with older adults, since it is small, portable, lightweight, equipped with motion detector and needs minimum involvement of the participant [61]. The reliability and spatial accuracy of this device is good in urban areas [61].

The tracking units were set on motion detector mode and 2-s recording interval. They were given to participants from low- and high-deprivation areas. Participants agreed to use the unit when they went out of their homes. They received oral and written instructions and were trained on how to wear the unit (on their wrists) and how to use it. Participants used the device for a period of 3 to 8 days (Mean = 4.95, SD = 1.61), depending on their willingness and availability. Each tracking unit was collected one day after the lending period and a questionnaire completed about its use. For example, each participant was asked if he/she used the unit; and how many days he/she forgot to use the unit. We excluded those who did not use/forgot to use the device.

GPS data provided by each participant were imported into a Geographic Information System (GIS) ArcGIS 10.3.1. (ESRI, Redlands, CA, USA). The recorded tracks were overlain with the road network and building footprints. For each participant, a “home-based neighbourhood”—a 2-km Euclidean buffer around the participant’s home, which is known as the most commonly used area for outdoor walking [62]—was defined. The participant’s outdoor walking—started from any point and ended in any point (e.g., walking from home to a park, walking around a block, walking from a car park/bus stop to a supermarket, walking in a park)—that took place within this area were taken into account (focusing on the “home-based neighbourhood” did not have considerable effects on recorded outdoor walking levels, see the Supplementary Materials Table S1). To measure outdoor walking level for each participant, first, daily outdoor walking activities were identified by using data on date and by applying criteria (on identifying walking trips using GPS data) suggested by Cho, et al. [63]. Then, durations of all daily outdoor walking activities were calculated by using data on time. Finally, (average) outdoor walking level (minutes per day) was calculated this way: (sum of duration of all daily outdoor walking activities)/(number of days that participant was loaned the GPS device).

### 2.4. Measuring Perceived Neighbourhood Built Environment Attributes

Two methods of data collection were used to measure how participants perceived neighbourhood built environment attributes: a questionnaire and a walking interview.

#### 2.4.1. Questionnaire

The Neighbourhood Environment Walkability Scale (NEWS) [64]—a questionnaire developed by Saelens, et al. [65]—was the basis for measuring perceived neighbourhood safety, pedestrian infrastructure and aesthetics. Some of the terminology was modified to be applicable for the UK participants (e.g., using “pavement” instead of “sidewalk”). Other elements of the questionnaire were modified to be applicable for older adults (e.g., adding items about width of pavements [65] and well-maintained front gardens [28]). According to definitions of neighbourhood built environment attributes (Table 1), different aspects of neighbourhood pedestrian infrastructure (e.g., traffic condition, pavement condition and amenities) were addressed. Therefore, five subscales (17 items) related to neighbourhood pedestrian infrastructure were included in the questionnaire (Table 2). Two subscales related to neighbourhood pedestrian infrastructure (i.e., presence of amenities and quietness) which were identified in literature on older adults’ walking [29,66], were added to the questionnaire. Quietness and air quality were included as single-item subscales related to pedestrian infrastructure, because they concern ambient stressors and are not subsumed under themes of other subscales (Table 2). Similar to the NEWS, one subscale (six items) related to neighbourhood safety and one subscale (6 items) related to neighbourhood aesthetics were included in the questionnaire (Table 2). All in all, seven subscales were included in the questionnaire (Table 2). The reliability testing showed acceptable internal consistency, Cronbach’s α > 0.65 [67], of each subscale (Table 2). NEWS questions which were irrelevant to safety, pedestrian infrastructure, aesthetics and walking were removed from the questionnaire. One NEWS item (i.e., “trees give shade for the sidewalks in my neighborhood”) was removed due to missing data and two NEWS items (i.e., “pavements are separated from the road/traffic in my neighborhood by parked cars”; and “there is a grass/dirt strip that separates the streets from the pavements in my neighborhood”) were removed in order to improve internal consistency of a subscale (i.e., pavement condition).

All items were rated on the 6-point Likert-type scale (ranging from (1) strongly disagree to (6) strongly agree). Comparing to 4-point Likert-type scale [68], which was used in original version of the NEWS, a 6-point Likert-type scale allows the use of parametric statistics for analyses [69] and also offers higher discriminating power, reliability and validity of scores [68]. All participants (*n* = 173) answered these closed questions during the GPS lending period and provided quantitative data on perceived neighbourhood safety, pedestrian infrastructure and aesthetics.

The subscales were scored according to the scoring protocol for the NEWS [65]; the total score of each subscale was the mean across the items of the subscale. Reverse coding was used where necessary; higher scores indicated a more favourable perception of neighbourhood safety, pedestrian infrastructure and aesthetics. Table 2 shows items, and total score of each subscale of the questionnaire.

Six personal characteristics were included in this study: age (65–74 years old or 75 years old and over); gender; marital status (single or in relationship); ethnicity (black and minority ethnic (BME) groups—including Asian, Black or mixed ethnic heritage—versus white British [70]); educational attainment (sub-GCSE (General Certificates of Secondary Education or its equivalents) or GCSE and higher); and perceived health status over the last twelve months (poor or good).

Missing data on each subscale and personal characteristic was less than 5% (except educational attainment with 11% missing data).

Open-ended survey questions: one open-ended question was added to each subscale of the NEWS questionnaire that was used for collecting quantitative data, for example asking “What are other issues related to safety in your neighbourhood? Please explain how the issues related to safety in your neighbourhood encourage or discourage you to walk outside your home”. All participants (*n* = 173) could answer these open-ended questions and explain their perceptions of their neighbourhoods in detail. They also could expand their responses to closed questions of the questionnaire and comment on related issues that were not mentioned in the questionnaire. Twenty-six participants from low-deprivation areas and twenty-six participants from high-deprivation areas answered the open-ended questions and provided qualitative data on perceived neighbourhood built environment (Table 3).

#### 2.4.2. Walking Interview

Walking interview provides rich qualitative data on perceptions of neighbourhood built environment [71,72]. Individual open-question walking interviews were conducted with participants from low- and high-deprivation areas (Table 3). Participants could speak English and were informed about the purpose of research. Participants were asked to select walking routes to take the interviewer around their neighbourhoods and to show positive and negative aspects of their neighbourhoods for walking. Through walking interviews, participants were enabled to provide more meaning and detail on their perceptions of neighbourhood safety, pedestrian infrastructure and aesthetics. They were asked to explain how their neighborhoods built environment supports, encourages or discourages them to take outdoor walks, providing detailed information about their preferences for taking outdoor walks and any challenges faced. They explained what makes outdoor walking dangerous, uncomfortable or enjoyable, where they do/do not walk and why, and they showed examples. The interviewer also asked follow-up questions about the issues mentioned by the participant. Depending on participants’ willingness, the interviews took between 30 to 60 minutes. A digital recorder and GPS tracking unit were used to record data.

### 2.5. Data Analysis

#### 2.5.1. Quantitative Analysis

Participants’ personal characteristics were analysed by using descriptive statistics. The spatial distributions of data on outdoor walking levels were analysed by applying ArcGIS 10.3.1 (ESRI, Redlands, CA, USA), using Natural Breaks in the data sets and classifying data in three levels (e.g., poor, neither poor nor good, and good).

Participants’ outdoor walking levels were compared between low- and high-deprivation areas by using independent sample *t*-test. Likewise, perceived neighbourhood built environment attributes were compared by area deprivation. These analyses were undertaken after finding an insignificant difference (*p* = 0.94) between average GPS lending period (number of days) in low- and high-deprivation areas (See Supplementary Materials Table S2).

To examine the relationships between perceived neighbourhood safety, pedestrian infrastructure and aesthetics, and outdoor walking levels, we used hierarchical linear regression analyses. Each perceived neighbourhood built environment attribute was examined individually. Each regression model was controlled for the interaction between perceived neighbourhood built environment attribute and area deprivation. When the interaction between the neighbourhood built environment attribute and area deprivation was related to outdoor walking levels, the analyses were undertaken for low- and high-deprivation areas separately. All regression models were controlled for participants’ marital status and ethnicity, because in this study, only these two personal characteristics were significantly associated with outdoor walking levels (participants who were in relationships or white British took outdoor walks more than single or BME groups, see Supplementary Materials: Tables S3–S6). In all regression models, missing data were excluded listwise. Moreover, logarithmic transformation on all variables were used to reduce heteroscedasticity. SPSS 22 (IBM, Armonk, NY, USA) was used for all statistical analysis and a *p*-value < 0.05 was considered as level of significance.

#### 2.5.2. Qualitative Analysis

Qualitative analysis focused on examining the same micro built environment attributes (i.e., perceived safety, pedestrian infrastructure and aesthetics) measured with quantitative tools in order to triangulate and corroborate [73] the quantitative finding. Therefore, a deductive approach was applied for the qualitative study. To ensure that important aspects of the qualitative data (the transcribed conversations of interviews and answers of open questions) were not missed, first, open coding was conducted [74]. Then, following a thematic analysis approach [75] three main themes (i.e., safety, pedestrian infrastructure and aesthetics) were defined. Afterwards, codes were linked to the themes. In this way, codes were categorised. To improve the reliability of analysis, the process was continued until data analysis reached saturation. The consistency of coding was rechecked by repeating the process [76]. All qualitative analyses were conducted using a computer Aided Qualitative Data Analysis (CAQDAS) software (ATLAS.ti Scientific Software Development GmbH, Berlin, Germany).

## 3. Results

### 3.1. Sample Characteristics

Sample characteristics are summarized in Table 4. This table shows that (in both total sample and total sub-sample) higher percentages of participants who were from BME groups or had low educational attainment (sub-GCSE) live in high-deprivation areas. Moreover, (in both total sample and total sub-sample) over 90 percent of participants living in both low- and high-deprivation areas perceived good health status.

### 3.2. Disparities in Participants’ Outdoor Walking Levels

**Figure 2:**
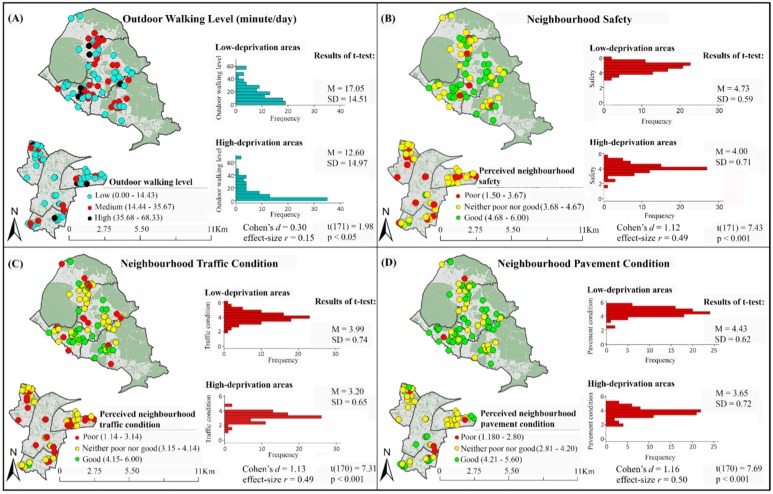

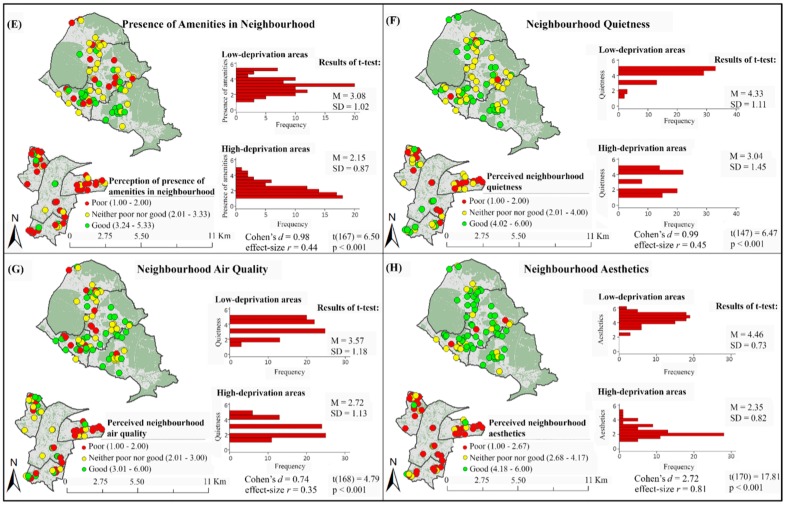
Disparities in outdoor walking levels and inequalities in perceived neighbourhood built environment attributes (OS open data Boundary-line © Crown copyright/database right 2012 and OS MasterMap data © Crown Copyright/database right 2012. An Ordnance Survey/EDINA Digimap supplied service). In all boxes of this figure, the image at the top shows low-deprivation areas and the image at the bottom shows high-deprivation areas. Frequency = number of participants; M = Mean; SD = Standard Deviation; *t* = *t* value.

Disparities in participants’ outdoor walking levels between low- and high-deprivation areas are presented in Figure 2A. This figure shows that high and medium outdoor walking levels are less prevalent in high-deprivation areas than in low-deprivation areas. The results of *t*-test also show that average outdoor walking level in high-deprivation areas is significantly lower than in low-deprivation areas (Figure 2A).

### 3.3. Spatial Inequalities in Perceived Neighbourhood Built Environment Attributes

Figure 2 presents inequalities in perceived neighbourhood safety, pedestrian infrastructure and aesthetics in low- and high-deprivation areas. As this figure shows, distributions of perceived good neighbourhood safety (Figure 2B) and good neighbourhood aesthetics (Figure 2H) are higher in low-deprivation areas than in high-deprivation areas. Similar trends were found for perceived neighbourhood pedestrian infrastructure (i.e., traffic condition (Figure 2C), pavement condition (Figure 2D), presence of amenities (Figure 2E), quietness (Figure 2F) and air quality (Figure 2G)). The results of the t-tests indicate that these differences are significant (Figure 2). Therefore, participants from high-deprivation areas perceived less safety, worse pedestrian infrastructure and lower aesthetic appeal in their neighbourhoods than those from low-deprivation areas.

### 3.4. Statistical Relationships between Perceived Neighbourhood Built Environment Attributes and Outdoor Walking Levels

Perceived neighbourhood safety, one aspect of pedestrian infrastructure (i.e., quietness) and aesthetics were significantly related to outdoor walking levels, before and after controlling for interactions between perceived neighbourhood built environment attributes and area deprivation (Table 5). Therefore, participants perceiving their neighbourhoods to be safer, quieter or more aesthetically pleasing are more likely to take longer outdoor walks. Perceived neighbourhood traffic condition, pavement condition, presence of amenities and air quality were not related to outdoor walking levels.

The interactions between area deprivation and some aspects of perceived neighbourhood pedestrian infrastructure (i.e., pavement condition, presence of amenities and air quality) were significantly related to outdoor walking levels (See Supplementary Materials Table S7). However, these aspects of perceived neighbourhood pedestrian infrastructure (i.e., pavement condition, presence of amenities and air quality) were not related to outdoor walking levels in low- and high-deprivation areas (Table 6). It means that relationships between perceived neighbourhood pavement condition, presence of amenities and air quality, and outdoor walking levels are similar in low- and high-deprivation areas and the results presented above (Table 5) are not moderated by area deprivation.

### 3.5. Qualitative Results on Perceived Influences of Neighbourhood Built Environment Attributes on Outdoor Walking Levels

Qualitative results provide further evidence on the perceived neighbourhood safety, pedestrian infrastructure and aesthetics and show to what extent these attributes may influence participants’ outdoor walking levels by supporting, encouraging or discouraging participants to take outdoor walks. The results show that participants from high-deprivation areas perceived more built environment constraints, especially in terms of safety, one aspect of pedestrian infrastructure (i.e., quietness) and aesthetics, which discouraged them from taking walks outside their homes. In the following sub-sections, qualitative results are explained in detail. All quotes presented in the following sub-sections are from walking interviews.

#### 3.5.1. Safety

Participants from both low- and high-deprivation areas explained that they do not take outdoor walks if they do not feel safe outside home. Participants from high-deprivation areas discussed lack of safety, especially for women, in their neighbourhoods. They explained that the (perceived) presence of gangs and groups of hooligans dominating pavements and their anti-social behaviour, as well as a high crime rate, drug use and lack of street lights make their neighbourhoods intimidating, unsupportive and discouraging for outdoor walking. A participant from a high-deprivation area said: “gangs hang around outside and you walk around not without their prejudice. You are not one of them (…) they won’t let you to pass through! They will come right up to you when you want to move!”. Participants perceived places with poor visibility to be more unsafe: “You wouldn’t know if there is anybody there or not, you can’t see!” (participant, high-deprivation area).

In contrast, participants from low-deprivation areas perceived safety in their neighbourhoods. One participant said: “I mean there is nothing to frighten anyone, there is no real crime in this area (New Oscott). You know, I mean there is no vandals around the streets. So, no frightening people”. Another participant explained that street lights provide good visibility and safety for walking in neighbourhood: “They’ve worked on it (lighting) mathematically. So that anywhere you walk, you can have this clear space in front of you to walk. I think it’s very good”.

In both low- and high-deprivation areas, presence of people in outdoor spaces and being watched or heard by other people were important for participants. Participants explained that they are concerned if they face an unexpected medical emergency (e.g., heart attack) when they are out and then need other people’s help. Moreover, being watched or heard was perceived as being important for safety from crime. One participant from a high-deprivation area said: “the point [of walking in a wide street] is that if anybody attacks to me, these houses are at far back, nobody can’t hear me”.

#### 3.5.2. Pedestrian Infrastructure

*Traffic condition*: participants from high-deprivation areas perceived more difficulties related to traffic conditions than those living in low-deprivation areas. In high-deprivation areas, perceived high traffic volume on busy roads (e.g., Coventry Road), high speed in quiet streets, lack of crossings (especially at close intervals), lack of traffic lights and presence of cars/lorries on pavements discouraged outdoor walking. Participants explained that to avoid traffic hazards, they take outdoor walks during quiet time of days or along quiet roads and they carefully cross the roads without using crossings. One participant said: “I confess I do go outside my house, but only in the quiet times. I look very carefully (for crossing the roads). I can say I stop half way, on the white lines you see in the middle of the road”. In low-deprivation areas, participants perceived ignoring speed limits and lack of traffic islands as main traffic hazards for outdoor walking. One participant noted: “It is easy (to cross the Mere Green Road) because you have good visibility both ways, but if cars are going sensibly!”. Some participants also reported about riding bicycles on pavements: “they haven’t run me over yet! But they might one day! Some bicycles don’t have bells. When they have bells you can hear them, but otherwise you can’t!” (participant, low-deprivation area).

*Pavement condition*: poor pavement condition and fear of falling were perceived as difficulties for outdoor walking in both low- and high-deprivation areas. Participants from low-deprivation areas explained that perceived narrow pavements and disturbance from infrastructure repairs (e.g., uneven paving slabs or manholes even after repair) in pavements make outdoor walking problematic. These difficulties were also expressed by participants from high-deprivation areas. As participants explained, trees/bushes from adjacent gardens and thick hedges make pavements narrower in low-deprivation areas and putting goods outside shops (e.g., in Alum Rock Road) make pavements narrow in high-deprivation areas. Participants from both low- and high-deprivation areas discussed problems caused by parked cars on pavements. Most participants, particularly in high-deprivation areas, also talked about perceived uneven pavements, broken slabs, presence of potholes, cracks and obstacles (e.g., knocked down bollards) in pavements. Participants carefully walked on these pavements: “when we walk up here, we have to be very careful! Broken and broken (pavements in Alum Rock Road)! This is disgusting! Broken pavements and slope! It would definitely mean you have to watch!” (participant, high-deprivation area). Participants said they choose even pavements for walking. They were concerned about falling on uneven pavements, being hurt and thus losing their confidence to walk outside. Moreover, a participant from a high-deprivation area noted that walking on an uneven pavement can exacerbate pain. She explained that uneven pavement disturbs the balance and if anybody suffers from foot problems and does not have right shoes, he/she will suffer from pain and will complain about the condition of pavement. As participants explained, walking on uneven pavements was perceived especially difficult when it is rainy and the pavements and potholes are flooded.

*Presence of amenities*: most participants from high-deprivation areas- as well as some participants from low-deprivation areas- reported lack of benches and public toilets in their neighbourhoods. Participants said they use benches and toilets of shops, malls or supermarkets. They explained that if they want to take longer walks, they may need more benches and public toilets: “If I want to do more (walking), I would need more places where I can rest and more toilets” (participant, high-deprivation area). As participants explained, public toilets in high-deprivation areas had been closed due to safety issues (e.g., crime and vandals). Although there were self-contained public toilets (superloo) in both low- and high-deprivation areas (e.g., in Boldmere and Small Heath) participants ignored them or said they do not use them because they perceived them complicated or not user-friendly. One participant from a high-deprivation area explained that it is difficult for him to use self-contained public toilets because he has to put money in it and he is illiterate. Another participant from a high-deprivation area also noted: “It’s not really a public one. Well, it’s like a weird thing! I do not use it”. Participants from low- and high-deprivation areas perceived presence of shelters (e.g., bus stops) in their neighbourhoods. One participant noted, however, that when it is rainy, a shelter is not useful if the pavement under the shelter is flooded.

*Quietness*: participants from both low- and high-deprivation areas explained that a quiet neighbourhood definitely encourages them to take outdoor walks. Participants from low-deprivation areas were satisfied with quietness of their neighbourhoods: “I like to walk in quiet streets definitely! There is no doubt about that, and this is quiet” (participant, low-deprivation area). However, participants from high-deprivation areas discussed noise, especially from traffic, in their neighbourhoods. Annoyance about noise dissuaded participants from going out and taking outdoor walks. One participant commented: “This is the area that I don’t like to walk. As I told you, there is a lot of noise”.

*Air quality*: a few participants from high-deprivation areas talked about low air quality of their neighbourhoods and explained that they prefer clean air for walking. A participant from a high-deprivation area pointed to different air qualities in different seasons: “You don’t see any smoke, but in the winter, you’ll see the smoke from the cars and form the lorries. But in the summer it is better. It’s bad in the winter”. Participants from low-deprivation areas, however, said good air quality of their neighbourhoods encouraged them to walk. One participant noted: “I like fresh air and exercise. This area, has a good air quality because of the (few) cars”.

#### 3.5.3. Aesthetics

Participants discussed the importance of the neighbourhood aesthetics for outdoor walking. Participants from high-deprivation areas discussed lack of beauty in their neighbourhoods. They explained that dirty streets and alleys, lack of beautiful natural sights (e.g., greenery and trees), lack of attractive houses/buildings, lack of front gardens and presence of industrial sites made their neighbourhoods boring, uninteresting and unenjoyable places which do not motivate them to go out and discouraged them from taking outdoor walks. One participant commented: “It (my neighbourhood) is boring. These little industries are around. There is not many pretty gardens and places to look up regularly. You can see all industries over there! Mixtures of old and new are round here, just the manufactory and industry, and what we call “back to back houses!” Participants explained that improving their neighbourhoods’ aesthetics (especially cleanness and greenery) would encourage them to take more outdoor walks: “I will walk more if they (authorities) put some flowers and something like that. I will be quite happy with things like that” (participant, high-deprivation area). In contrast, participants from low-deprivation areas were proud of their neighbourhoods’ aesthetics. They explained that cleanness, presence of greenery, presence of nice natural landscape, well-maintained gardens and attractive buildings of their neighbourhoods provided beautiful and enjoyable areas and gave them lots of incentives for taking outdoor walks: “We’ve got lots of trees and bushes and (…) green grass verges, so it is (a good place for walking)!” (participant, low-deprivation area). From these participants’ perspectives, the presence of large beautiful green spaces (e.g., Sutton Park and New Hall Valley) is one of the reasons why they have nice natural sights in their neighbourhoods.

### 3.6. Combining Quantitative and Qualitative Results

Table 7 shows a combination of quantitative and qualitative results. Quantitative results show that perceived neighbourhood safety, one aspect of pedestrian infrastructure (i.e., quietness) and aesthetics are positively related to outdoor walking levels. Qualitative findings support these quantitative results, showing that from participants’ perspective, safe neighbourhoods, good neighbourhood pedestrian infrastructure and aesthetically pleasurable neighbourhoods encourage outdoor walking. Quantitative results also show spatial inequalities in perceived neighbourhood safety, pedestrian infrastructure (i.e., quietness) and aesthetics in high- versus low-deprivation areas. Qualitative results support these findings and extend them by showing poorer provision of safety, quietness and built and natural attractiveness in high- versus low-deprivation areas. Combining the quantitative and qualitative results indicates that inequalities in perceived neighbourhood safety, one aspect of pedestrian infrastructure (i.e., quietness) and aesthetics in high- versus low-deprivation areas may influence the disparities in participants’ outdoor walking levels. Inequalities were also found in other aspects of perceived neighbourhood pedestrian infrastructure (i.e., traffic condition, pavement condition, presence of amenities and air quality) in high- versus low-deprivation areas. However, these inequalities do not influence the disparities in participants’ outdoor walking levels between these areas, because these aspects of perceived neighbourhood pedestrian infrastructure are not significantly related to participants’ outdoor walking levels.

## 4. Discussion

The aim of this study was to examine spatial inequalities in perceived neighbourhood safety, pedestrian infrastructure and aesthetics in high- versus low-deprivation areas and their possible influences on disparities in older residents’ total outdoor walking levels in Birmingham, UK. In line with previous studies [8], this study found that participants from high-deprivation areas walk in outdoor spaces less than those from low-deprivation areas. This study showed that inequalities in perceived neighbourhood safety, one aspect of pedestrian infrastructure (i.e., quietness) and aesthetics in high- versus low-deprivation areas may influence disparities in participants’ total outdoor walking levels between these areas. The findings of this study are discussed in the following sub-sections.

### 4.1. Perceived Neighbourhood Safety, Quietness and Aesthetics

Perceived neighbourhood safety, quietness (as an aspect of neighbourhood pedestrian infrastructure) and aesthetics were positively related to total outdoor walking levels. These findings support evidence indicating that older adults’ walking level is related to neighbourhood built environment [11,12,13]. They are also consistent with previous quantitative research showing positive relationships between perceived neighbourhood safety [12,77] and aesthetics [78], and walking levels among older adults. They also support previous qualitative research indicating that perceived neighbourhood safety, quietness and aesthetics encourage outdoor walking among older adults [66,79,80,81].

In this study, the relationships between outdoor walking levels and perceived neighbourhood safety, quietness (as an aspect of neighbourhood pedestrian infrastructure) and aesthetics were not moderated by area deprivation. It means that relationships between perceived neighbourhood safety, quietness and aesthetics, and outdoor walking levels are similar for low- and high-deprivation areas. Qualitative results of this study also showed that perceived neighbourhood built environment attributes (i.e., safety, pedestrian infrastructure and aesthetics) were important for outdoor walking in both low- and high-deprivation areas.

This study found spatial inequalities in perceived neighbourhood safety, quietness and aesthetics, which were related to outdoor walking levels, in high- versus low-deprivation areas. Identifying spatial inequalities in perceived neighbourhood safety was an expected result, since the crime domain has been included in the IMD score used for identifying low- and high-deprivation areas [49]. Findings on spatial inequalities in perceived neighbourhood safety and aesthetics are consistent with findings of previous research examining adults’ physical activity [41,82,83] and older adults’ leisure time physical activity [84] in low- and high-deprivation areas. Qualitative findings of this study supported quantitative findings and showed that lack of safety, good pedestrian infrastructure (e.g., quietness) and aesthetics discourage outdoor walking in high-deprivation areas.

### 4.2. Other Aspects of Perceived Neighbourhood Pedestrian Infrastructure

In this study, perceived traffic condition and pavement condition, as two aspects of neighbourhood pedestrian infrastructure, were not statistically related to outdoor walking levels. Perceived traffic condition, however, is probably correlated with perceived quietness (which is related to outdoor walking levels): qualitative findings showed that traffic noise affects participants’ perceptions of quietness in their neighbourhood (correlations between perceived neighbourhood built environment attributes were also tested quantitatively and reported in the Supplementary Materials Table S8). The non-significant relationships between perceived traffic condition and pavement condition, and outdoor walking levels may be explained by health status in the sample. It is likely that the good health status of the majority of participants helped them to deal with perceived traffic and falling hazards in their neighbourhoods. Consistent with previous research [28,29], qualitative results showed that perceived traffic condition and pavement condition are important for outdoor walking. These results also showed that participants, from both low- and high-deprivation areas, perceived unfavourable traffic and pavement conditions that made outdoor walking uncomfortable for them (Table 7), but participants had strategies to cope with and overcome these difficulties (e.g., they carefully crossed the roads without using crossings). It was also found that walking on uneven pavements is especially difficult for people who suffer from pain or people with severe mobility problems. Therefore, these two aspects of perceived neighbourhood pedestrian infrastructure (i.e., traffic condition and pavement condition) may be significantly related to outdoor walking levels among older adults with poorer health status. Future research may investigate this issue by using a more heterogeneous sample in terms of health status.

Perceived presence of amenities and air quality were two other aspects of neighbourhood pedestrian infrastructure that were not statistically related to outdoor walking levels. These neighbourhood built environment attributes have been less addressed as subscales in previous quantitative studies on older adults’ walking. The non-significant relationships between perceived presence of amenities and air quality, and outdoor walking levels may be explained by qualitative findings. In terms of amenities, as qualitative results showed, participants from both low- and high-deprivation areas perceived lack of benches and public toilets in their neighbourhoods (Table 7), but they had other alternatives to use (e.g., benches and public toilets in malls). Therefore, lack of amenities in neighbourhood did not discourage participants from taking outdoor walks. Qualitative findings also showed that participants from both low- and high-deprivation areas had the same belief that fresh air encourages them to take outdoor walks (Table 7). However, participants perceived poorer air quality in winter. Since the air quality item of the questionnaire was not about a specific season, it is likely that participants took general air quality (in summer and winter) of their neighbourhoods into account when they filled the questionnaires. If this assumption holds true, perceived general air quality is not related to participants’ outdoor walking levels in a specific season (i.e., summer) when the study was conducted. Future studies may help to improve knowledge on this issue by including items on neighbourhood air quality in different seasons in the questionnaire. Qualitative findings of this study confirm previous qualitative research showing that amenities (i.e., toilets and benches) facilitate [29,66] and clean air encourages outdoor walking among older adults [66].

### 4.3. Application of the Findings

The built environment may influence behaviour by influencing people’s perception [85]. As Lawton and Nahemow [86] have argued, older adults’ behavior (e.g., outdoor walking) is dependent on the adaptation between persons’ abilities and the built environment demands. Urban planners need to understand person-built environment interactions, because they intend to provide supportive built environment for older adults’ outdoor walking. Although perceived built environment may not reflect the actual built environment, it helps urban planners to identify the functional qualities of the actual built environment. Urban planners need to improve their knowledge on qualities of the built environment before modifying the actual built environment. Therefore, it may be useful to conduct surveys on influences of built environment attributes on outdoor walking levels before developing urban areas.

The findings of this study on perceived neighbourhood built environment attributes may help urban planners to understand how the neighbourhood built environment they have planned interacts with residents. These findings are useful in recognising the gaps that exist between older adults’ abilities and neighbourhood built environment, helping professionals to coordinate remedial strategies and to create new built environmental opportunities to encourage participants to take more outdoor walks.

This study also reflects the experience of older adults living in different circumstances. It warns policy makers not to enlarge the spatial inequalities by contriving interventions that support outdoor walking only in low-deprivation areas (and not in high-deprivation areas). The findings of this study indicate that improving neighbourhood safety, quietness and aesthetics may encourage participants living in high-deprivation areas to take more outdoor walks. Considering correlations between these perceived neighbourhood built environment attributes may help to decide about interventions priorities. For example, if perceived neighbourhood safety and aesthetics are highly correlated, improving perceived aesthetics (e.g., fixing broken windows or cleaning streets) may also improve perceived neighbourhood safety [22].

Perception of neighbourhood safety, quietness and aesthetics may be improved by modifying the actual built environment (e.g., improving perceived neighbourhood safety through installing street lights and providing more windows overlooking the street [87]) or without modifying the actual built environment (e.g., improving perceived neighbourhood safety through investing much greater police effort in neighbourhood [88]). Therefore, to improve the positive perceptions of neighbourhood safety, quietness and aesthetics, involving urban planning professionals as well as other professionals (e.g., from social sector, urban security sector, traffic management and maintenance) may be required.

### 4.4. Limitations

This study has some limitations. It is a cross-sectional study, so it does not make a causal inference. Moreover, it found inequalities in perceived neighbourhood built environment attributes, but it is unknown whether these inequalities reflect actual differences in the built environment between high- and low-deprivation areas. Examining actual neighbourhood built environment attributes in high- and low-deprivation areas is also important. Linking actual and perceived inequalities in neighbourhood built environment attributes may help to identify how to improve positive perception of neighbourhood safety, quietness and aesthetics in high-deprivation areas. Future studies may investigate actual inequalities in neighbourhood built environment attributes in high- versus low-deprivation areas by using objective data (e.g., on level of traffic noise and areas of green spaces).

This study was done in a single UK city and used a convenience sample of older adults from social centres. These participants might not be representative of all older adults, especially those with poor health status, living in low- and high-deprivation areas. Notwithstanding these limitations, this study offers an insight into the spatial inequalities in low- and high-deprivation areas, which is applicable to more heterogeneous samples, other places and future studies.

## 5. Conclusions

This study improves knowledge on perceived attributes of the built environment encouraging citizens (i.e., older adults) to take outdoor walks. It also adds to the growing body of knowledge concerning the influences of spatial inequalities in the built environment (in high- versus low-deprivation areas) on physical activity levels. This study is among the first research on older adults’ total outdoor walking using a spatial inequality approach and GPS technology. It showed that spatial inequalities in perceived neighbourhood safety, one aspect of pedestrian infrastructure (i.e., quietness) and aesthetics in high- versus low-deprivation areas may influence the disparities in participants’ outdoor walking levels between these areas. Therefore, participants from high-deprivation areas perceived less neighbourhood support for outdoor walking than those from low-deprivation areas. Improving living environment conditions leading to positive perception of neighbourhood safety, pedestrian infrastructure and aesthetics in high-deprivation areas is encouraged. 

## Figures and Tables

**Table 1:** Definitions of neighbourhood safety, pedestrian infrastructure and aesthetics.

Neighbourhood Built Environment Attribute	Definition
Safety	Refers to the relative absence of threat from crime to residents of a neighbourhood. A safe neighbourhood offers less fear of crime by providing built environmental elements (e.g., street lights or windows in ground floor of buildings) and lower crime rate [31].
Pedestrian infrastructure	Refers to the traffic and pavement conditions (e.g., traffic calming elements and pavement maintenance) and amenities (e.g., benches) that facilitate walking in a neighbourhood. A good pedestrian infrastructure make walking comfortable by reducing traffic hazards and risks of falling, providing amenities [31] and decreasing ambient stressors (e.g., noise).
Aesthetics	Refers to a sense of beauty and visual appearance of a neighbourhood. An aesthetically pleasurable neighbourhood invites resident to walk by offering an enjoyable area for walking and providing built and natural attractiveness (e.g., presence of attractive architecture and trees) [31].

**Table 2:** Description of subscales of the questionnaire.

Subscale	Number of Items	Content Description	Cronbach Alpha (α)	M (SD) ^a^
Safety	6	My neighbourhood streets are well lit/Pedestrian can be easily seen by people in their homes/I see and speak to other people when I walk in my neighbourhood/there is a high crime rate/the crime rate makes it unsafe to walk during day/the crime rate makes it unsafe to walk in the evening	0.73	4.39 (0.74)
Traffic condition	7	There is so much traffic along the street I live in/there is high speed of traffic on the street I live/there is so much traffic along the nearby streets/The speed of traffic is slow on the nearby streets/drivers exceed the speed limit in neighbourhood/there are crosswalks and pedestrian signals to help walkers/crosswalks help to feel safe crossing busy streets	0.71	3.62 (0.80)
Pavement condition	5	There are pavements on most streets/pavements are well-maintained/pavements are wide enough/clutters (e.g., poles, bollards, etc.) in pavements make walking difficult/vehicle park on pavement, leaving too little space for walking	0.65	4.07 (0.77)
Presence of amenities	3	There are shelters (e.g., bus stop) protecting me from rain and wind/ there are adequate public seating or benches and I can rest whenever I feel tired/there are adequate public toilets and I can easily use them	0.72	2.64 (1.05)
Quietness	1	My neighbourhood is quiet and noiseless	---	3.73 (1.43)
Air quality	1	There are a lot of exhaust fumes	---	3.18 (1.23)
Aesthetics	6	There are trees along the streets/there are many interesting things to look at/my neighbourhood is free from litter/there are many attractive natural sights/well-maintained front gardens have created attractive streets/there are attractive buildings in my neighbourhood	0.92	3.48 (1.31)

Note: Five subscales (i.e., traffic condition, pavement condition, presence of amenities, quietness, and air quality) are related to neighbourhood pedestrian infrastructure. ^a^ response score: (1) strongly disagree and (6) strongly agree, M = Mean of total scores; SD = Standard deviation of total scores.

**Table 3:** Detail information about participants who provided qualitative data.

Participants’ Characteristics	Walking Interview			Open-Ended Questions		
	Low	High	Total	Low	High	Total
Number of participants	9	10	19	26	26	52
Age (*n*):						
75 years old and over	5	5	10	16	13	29
65–74 years old	4	5	9	10	13	23
Gender (*n*):						
Men	2	4	6	6	11	17
Women	7	6	13	20	15	35
Marital status (*n*):						
In relationship	6	5	11	15	11	26
Single	3	5	8	11	15	26
Ethnicity (*n*):						
White British	8	5	13	26	12	38
BME groups	1	5	6	0	14	14
Educational attainment (*n*):						
GCSE and higher	9	2	11	21	9	30
Sub-GCSE	0	8	8	2	13	15
Health status (*n*):						
Good	9	9	18	24	24	48
Poor	0	1	1	2	2	4

Note: Walking interview: participants who participated in walking interview, Open-ended questions = participants who completed open ended questions, Low = sample from low-deprivation areas, High = sample from high-deprivation areas, Total = sample from both low- and high-deprivation areas, *n* = number.

**Table 4:** Sample characteristics in low- and high-deprivation areas and in total.

Participants’ Characteristics	Total Sample			Total Sub-Sample		
	Low	High	Total	Low	High	Total
Number of participants	93	80	173	35	36	71
Average age of participants (M (SD))	74.8 (5.82)	73.5 (5.95)	74.2 (5.90)	75.46 (6.09)	73.00 (6.33)	74.2 (6.29)
Age (%):						
75 years old and over	53	43	48	60	50	55
65–74 years old	47	57	52	40	50	45
Gender (%):						
Men	30	59	43	23	42	32
Women	70	41	57	77	58	68
Marital status (%):						
In relationship	53	53	53	60	44	52
Single	47	47	47	40	56	48
Ethnicity (%):						
White British	97	41	71	97	47	72
BME groups	3	59	29	3	53	28
Educational attainment (%):						
GCSE and higher	80	24	54	86	31	58
Sub-GCSE	10	64	35	6	58	32
Health status (%):						
Good	93	92	92	94	92	94
Poor	6	8	7	6	8	7

Note: Total sample = the whole sample used for quantitative study, Total sub-sample = the whole sample used for qualitative study (both walking interview sample and sample who completed open-ended questions in the questionnaire), Low = sample from low-deprivation areas, High = sample from high-deprivation areas, Total = sample from both low- and high-deprivation areas, M = Mean, SD = Standard Deviation.

**Table 5:** Results of hierarchical regression analyses: relationships between perceived neighbourhood built environment attributes and outdoor walking levels.

Perceived Neighbourhood Built Environment Attribute	Outdoor Walking Levels	
	Before Controlling for Interaction ^a^	After Controlling for Interaction ^a^
	*B (SE)*	*B (SE)*
Safety	1.33 (0.48) **	1.20 (0.49) *
Traffic condition	0.48 (0.37)	0.37 (0.38)
Pavement condition	0.06 (0.43)	−0.10 (0.43)
Presence of amenities	0.33 (0.23)	0.40 (0.23)
Quietness	0.54 (0.17) **	0.57 (0.18) **
Air Quality	0.16 (0.20)	0.28 (0.21)
Aesthetics	0.55 (0.22) *	0.53 (0.23) *

Note: Traffic condition, pavement condition, presence of amenities, quietness, and air quality are five aspects of perceived neighbourhood pedestrian infrastructure. Each perceived neighbourhood built environment attribute was examined individually. This table shows the results after controlling for personal characteristics (i.e., marital status and ethnicity). ^a^ Interaction between each perceived neighbourhood built environment attribute and area deprivation. *B* = Unstandardised Coefficient; *SE* = Standard Error. The values in **bold** type are significant. * *p* < 0.05, ** *p* < 0.01.

**Table 6:** Results of hierarchical regression analyses: relationships between three aspects of perceived neighbourhood pedestrian infrastructure and outdoor walking levels in low- and high-deprivation areas.

Aspects of Perceived Neighbourhood Pedestrian Infrastructure	Outdoor Walking Levels	
	Low-Deprivation Areas	High-Deprivation Areas
	*B (SE)*	*B (SE)*
Pavement condition	−0.69 (0.71)	−0.16 (0.63)
Presence of amenities	0.58 (0.30)	−0.43 (0.42)
Air quality	0.15 (0.29)	−0.08 (0.31)

Note: This table shows the results after controlling for personal characteristics (i.e., marital status and ethnicity). *B* = Unstandardised Coefficient; *SE* = Standard Error.

**Table 7:** Combination of quantitative and qualitative results.

Neighbourhood Built Environment Attribute	Quantitative Results		Qualitative Results
	Spatial Inequalities	Related to Walking Levels	Perceived Influences of Neighbourhood Built Environment Attributes on Outdoor Walking Level
Safety	High < Low	Yes	High: perceived intimidating neighbourhoods were unsupportive and discouraging for outdoor walking. Low: perceived safe neighbourhoods supported and encouraged participants to take outdoor walks.
Traffic condition	High < Low	No	High: perceived poor traffic conditions made outdoor walking uncomfortable. Participants took outdoor walk in quiet traffic time or in quiet roads and they carefully crossed the roads without using crossings. Low: perceived some poor traffic conditions made walking uncomfortable.
Pavement condition	High < Low	No	High and Low: perceived poor pavement conditions made walking uncomfortable. Participants chose even pavements for walking.
Presence of amenities	High < Low	No	High and Low: lack of benches and public toilets were perceived. Participants used benches and public toilets of shops, malls and supermarkets.
Quietness	High < Low	Yes	High: perceived noise, especially from traffic, in neighbourhood dissuaded participants to walk outside. Low: perceived quietness of neighbourhood definitely encouraged outdoor walking.
Air quality	High < Low	No	High: air quality was perceived poorer in winter. A clean air was preferred for outdoor walking. Low: perceived clean air encouraged outdoor walking.
Aesthetics	High < Low	Yes	High: perceived boring, uninteresting and unenjoyable neighbourhoods discouraged outdoor walking. Low: perceived beautiful and enjoyable neighbourhoods encouraged outdoor walking.

Note: Traffic condition, pavement condition, presence of amenities, quietness, and air quality are five aspects of neighbourhood pedestrian infrastructure. Low = low-deprivation areas; High= high-deprivation areas.

## Supplementary Materials

The following are available online at www.mdpi.com/1660-4601/13/12/1179/s1, Table S1: Difference between outdoor walking levels in ‘city’ and in ‘home based neighbourhood’, Table S2: Difference between GPS lending period in low- and high-deprivation areas, Table S3: Correlations between personal characteristics and perceived neighbourhood built environment attributes, Table S4: Results of hierarchical regression analyses: relationships between outdoor walking levels and personal characteristics, Table S5: Results of hierarchical regression analyses: relationships between outdoor walking levels and interactions between personal characteristics and area deprivation, Table S6: Results of hierarchical regression analyses: relationships between personal characteristics and outdoor walking levels in low- and high-deprivation areas, Table S7: Results of hierarchical regression analyses: relationships between outdoor walking levels and interactions between perceived neighbourhood built environment attributes and area deprivation; Table S8. Correlations between perceived neighbourhood built environment attributes.
