# Supplementary material for: Older Adults’ Outdoor Walking: Inequalities in Neighbourhood Safety, Pedestrian Infrastructure and Aesthetics
Source: Int J Environ Res Public Health. 2016 Nov 25;13(12):1179. doi: 10.3390/ijerph13121179 (PMC5201320; doi:10.3390/ijerph13121179)
Supplement: Supplementary file 1 [file ijerph-13-01179-s001.pdf]

# Supplementary Materials: Older Adults' Outdoor Walking: Inequalities in Neighbourhood Safety, Pedestrian Infrastructure and Aesthetics

**Razieh Zandieh, Javier Martinez, Johannes Flacke, Phil Jones and Martin van Maarseveen**

**Table S1.** Difference between outdoor walking levels in "city" and in "home based neighbourhood".

| Outdoor Space | Outdoor Walking Levels | | |
|---|---|---|---|
| | ***M*** | ***SD*** | ***t*-Value** |
| City | 17.30 | 16.35 | $t$ (172) = 1.68; |
| Home based neighbourhood | 14.99 | 14.85 | $p$ = 0.094 |

Note: This table shows the results of paired *t*-test used for comparing outdoor walking levels in "city" and in "home based neighbourhood". As this table shows, although (average) outdoor walking levels in "city" is higher than (average) outdoor walking levels in "home based neighbourhood", this difference is not significant. Therefore, focusing on the "home-based neighbourhood" does not have considerable effects on recorded outdoor walking levels. *M* = Mean; *SD* = Standard Deviation.

**Table S2.** Difference between GPS lending period in low- and high-deprivation areas.

| Area Deprivation | GPS Lending Period (Number of Days) | | |
|---|---|---|---|
| | ***M*** | ***SD*** | ***t*-Value** |
| Low | 4.96 | 1.47 | $t$ (154) = 0.08, $p$ = 0.94 |
| High | 4.94 | 1.78 | |

Note. *M* = Mean; *SD* = Standard Deviation.

**Table S3.** Correlations between personal characteristics and perceived neighbourhood built environment attributes.

| Personal Characteristics | Ethnicity | Safety | Traffic Condition | Pavement Condition | Presence of Amenities | Quietness | Air Quality | Aesthetics |
|---|---|---|---|---|---|---|---|---|
| Marital status | −0.09 (0.216) | 0.17 (0.027) | 0.09 (0.259) | 0.03 (0.703) | −0.26 (0.001) | 0.30 (0.694) | 0.09 (0.258) | 0.01 (0.914) |
| Ethnicity | | 0.23 (0.002) | 0.22 (0.003) | 0.27 (0.000) | 0.33 (0.000) | 0.15 (0.023) | 0.17 (0.027) | 0.412 (0.000) |

Note: This table shows Pearson correlation values; *p*-values are presented in parentheses. In this study, Likert scale results for each subscale were aggregated. As this table shows, there are weak correlations between personal characteristics and (aggregated) perceived neighbourhood built environment attributes. We tested if the aggregation method influenced the weak correlations between personal characteristics (i.e., marital status and ethnicity) and perceived neighbourhood built environment attributes (Table S3). For this purpose, the correlations between each item of the questionnaire and personal characteristics and correlations between each item of the questionnaire and outdoor walking levels were tested. Pearson correlation values showed the maximum correlation between one item (i.e., there are many attractive natural sights) and ethnicity ($r_{max}(171) = −0.41$, $p = 0.000$). Therefore, the influence of aggregation method on weak correlations between personal characteristics and perceived neighbourhood built environment attributes was not considerable.

**Table S4.** Results of hierarchical regression analyses: relationships between outdoor walking levels and personal characteristics.

| Personal Characteristics | Outdoor Walking Levels | |
|---|---|---|
| | ***B*** | ***SE*** |
| Marital status | 0.79 ** | 0.25 ** |
| Ethnicity | 0.59 * | 0.28 * |

Note: Relationships between all personal characteristics (i.e., age, gender, marital status, ethnicity, educational attainment and health status) and outdoor walking levels were examined by using linear regression models. Logarithmic transformed variables ($x + 1$) were used to reduce heteroscedasticity. All personal characteristics were entered at once in the model and then the least significant (in terms of *t*-value) variables were dropped in order to get the model of best fit. This table shows the results after dropping least significant predictors (i.e., age, gender, educational attainment and health status). *B* = Unstandardised Coefficient; *SE* = Standard Error. * $p < 0.05$, ** $p < 0.01$.

**Table S5.** Results of hierarchical regression analyses: relationships between outdoor walking levels and interactions between personal characteristics and area deprivation.

| Interaction [a] | Outdoor Walking Levels | |
|---|---|---|
| | ***B*** | ***SE*** |
| Model 1: | | |
| Marital status × area deprivation | −1.13 | 0.61 |
| Ethnicity × area deprivation | **−1.04 *** | **0.49 *** |
| Model 2: | | |
| Marital status × area deprivation | −0.58 | 0.73 |
| Ethnicity × area deprivation | −0.77 | 0.59 |

Note: [a] Interaction between a personal characteristic and area deprivation. Model 1: in addition to personal characteristics (i.e., marital status and ethnicity), interactions between personal characteristics and area deprivation were (separately) added to the model. Model 2: in addition to personal characteristics (i.e., marital status and ethnicity), a combination of two interactions between personal characteristics and area deprivation was added to the model. *B* = Unstandardised Coefficient; *SE* = Standard Error. The values in **bold** type are significant. * $p < 0.05$.

**Table S6.** Results of hierarchical regression analyses: relationships between personal characteristics and outdoor walking levels in low- and high-deprivation areas.

| Personal Characteristics | Outdoor Walking Levels | |
|---|---|---|
| | **Low-Deprivation Areas** | **High-Deprivation Areas** |
| | ***B (SE)*** | ***B (SE)*** |
| Marital status | **0.91 (0.32) **** | 0.48 (0.42) |
| Ethnicity | 0.84 (0.90) | −0.02 (0.42) |

Note: *B* = Unstandardised Coefficient; *SE* = Standard Error. The values in **bold** type are significant. ** $p < 0.01$.

**Table S7.** Results of hierarchical regression analyses: relationships between outdoor walking levels and interactions between perceived neighbourhood built environment attributes and area deprivation.

| Interaction [a] | Outdoor Walking Levels | |
|---|---|---|
| | *B* | *SE* |
| Safety × area deprivation | −0.27 | 0.23 |
| Traffic condition × area deprivation | −0.44 | 0.28 |
| Pavement condition × area deprivation | **−0.49 *** | **0.25 *** |
| Presence of amenities × area deprivation | **−0.79 *** | **0.34 *** |
| Quietness × area deprivation | −0.19 | 0.25 |
| Air quality × area deprivation | **−0.59 *** | **0.29 *** |
| Aesthetics × area deprivation | −0.12 | 0.35 |

Note: [a] Interaction between a perceived neighbourhood built environment attribute and area deprivation. Traffic condition, pavement condition, presence of amenities, quietness, and air quality are five aspects of perceived neighbourhood pedestrian infrastructure. Each interaction between perceived neighbourhood built environment attributes and area deprivation was examined individually. This table shows the result after adjustment for the perceived neighbourhood built environment attribute and personal characteristics (i.e., marital status and ethnicity) in each model. Relationships between perceived neighbourhood pavement condition, presence of amenities and air quality, and outdoor walking level were examined for low- and high-deprivation areas separately and the results were presented in the manuscript (Table 6). *B* = Unstandardised Coefficient; *SE* = Standard Error. The values in **bold** type are significant, * $p < 0.05$.

**Table S8.** Correlations between perceived neighbourhood built environment attributes.

| Perceived Neighbourhood Built Environment Attribute | Safety | Traffic Condition | Pavement Condition | Presence of Amenities | Quietness | Air Quality | Aesthetics |
|---|---|---|---|---|---|---|---|
| Safety | | 0.55 (0.000) | 0.38 (0.000) | 0.17 (0.23) | 0.43 (0.000) | 0.31 (0.000) | 0.47 (0.000) |
| Traffic condition | | | 0.54 (0.000) | 0.31 (0.000) | 0.41 (0.000) | 0.41 (0.000) | 0.58 (0.000) |
| Pavement condition | | | | 0.21 (0.006) | 0.46 (0.000) | 0.45 (0.000) | 0.52 (0.000) |
| Presence of amenities | | | | | 0.21 (0.006) | 0.10 (0.178) | 0.43 (0.000) |
| Quietness | | | | | | 0.40 (0.000) | 0.52 (0.000) |
| Air quality | | | | | | | 0.41 (0.000) |

Note: Traffic condition, pavement condition, presence of amenities, quietness, and air quality are five aspects of perceived neighbourhood pedestrian infrastructure. This table shows Pearson correlation values; *p*-values are presented in parentheses.
